# Dataset on the evaluation of antimicrobial activity and optical properties of green synthesized silver and its allied bimetallic nanoparticles

**DOI:** 10.1016/j.dib.2018.10.054

**Published:** 2018-10-24

**Authors:** Anuoluwa Abimbola Akinsiku, Enock Olugbenga Dare, Olayinka Oyewale Ajani, Joseph Adeyemi Adekoya, Alaba Oladipupo Adeyemi, Oluwaseun Ejilude, Kehinde David Oyeyemi

**Affiliations:** a Department of Chemistry, Covenant University, Nigeria; b Department of Chemistry, Federal University of Agriculture Abeokuta, Nigeria; c Faculty of Chemistry and Pharmacy, Institute for Organic Chemistry, Regensburg University, 31, Regensburg 93053, Germany; d Department of Chemistry, University of Zululand, Kwa-Dlangezwa, South Africa; e Department of Biochemistry, Covenant University, 1023, Ota, Nigeria; f Department of Medical and Parasitology, Sacred Heart Hospitals, Abeokuta, Nigeria; g Department of Physics, Covenant University, Nigeria

## Abstract

The pursuit for bioremediation has led to alternative route for the synthesis of nanoparticles and their hybrids. Data in this article display optical properties and progress in the formation of silver and silver/nickel bimetallic nanoparticles using eco-friendly reducing agent (Akinsiku et al. 2018). The as-prepared nanoparticles portrayed nanocrystalline nature as revealed in the x-ray powder diffraction (XRPD) data. Data also exposed antimicrobial activity of the synthesized nanoparticles.

**Specifications table**

**Table:** 

Subject area	Material chemistry, nano chemistry
More specific subject area	Ag and Ag/Ni bimetallic nanoparticles from plant biomass
Type of data	Table and Image (Generated)
How data was acquired	1. Optical measurements were carried out by double beam Thermo Scientific GENESYS 10S UV–vis spectrophotometer, at Covenant University, Canaan Land, Nigeria. 2. UV–vis spectrophotometer model T90+, at Sacred Heart Hospital, Abeokuta, Nigeria. 3. Bruker D8 XRD model, iThemba Labs, South Africa.
Data format	Raw and Analyzed
Experimental factors	Reduction of precursor with *Canna indica* leaf extract, heating to 70 °C on a hot plate for 30 min.
Experimental features	Optical property of the nanoparticles, evaluation of antimicrobial activity [1], crystallinity of the nanocluster.
Data source location	Plants were collected at Atan-Iju, Ogun State, Nigeria (Fig. 3).
Data accessibility	Dataset are available within this article.

**Value of the data**

• The data presented is from innovative research that provides additional eco-friendly and sustainable method for other researchers.
• The data could be useful for further studies in developing antibacterial drugs.
• These data could serve as an analytical tool for screening plants that are rich in vitamins and phytochemicals.
• These data could form a benchmark for other researchers who want to develop plant-mediated green nanoparticles.
• The data highlighted several moderate anti-bacterial effects of the as-prepared hybrid nanoparticles.

## 1 Data

### 1.1 Optical properties of Ag and Ag/Ni bimetallic nanoparticles

**Fig. 1:**
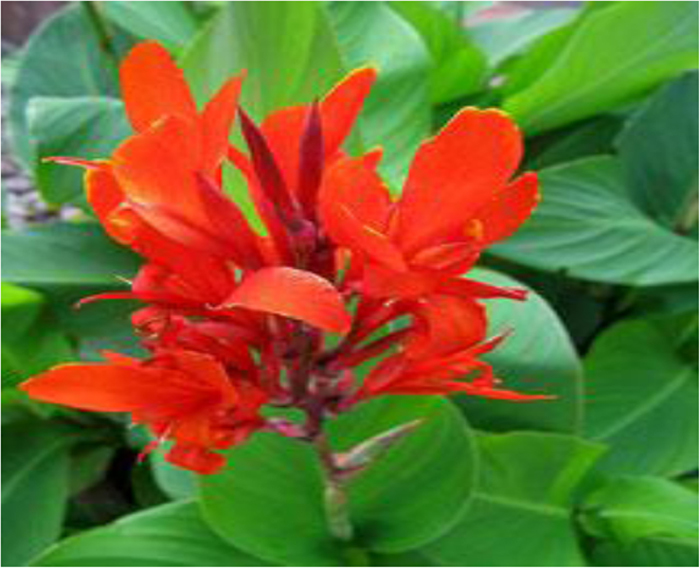
Image of the utilized *Canna indica* (FHI No. 109928).

**Fig. 2:**
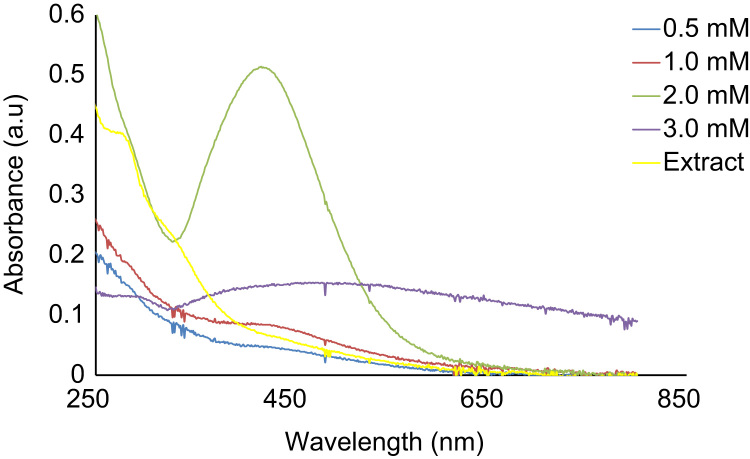
Growth comparison in Ag/Ni bimetallic nanoparticles using the extract of *C. indica* at 70 °C, 30 min.

Fig. 1 is image of the utilized plant: *Canna indica* with FHI No. 109928. It was identified and authenticated at FRIN herbarium headquarters, Ibadan, Nigeria. Voucher specimen of the plant was also deposited. Fig. 2 shows optical properties and growth comparison in Ag/Ni bimetallic nanoparticles using the extract of *C. indica* at 70 °C, 30 min, (See data in Supplementary Table 1).

### 1.2 Antimicrobial analysis

Similar activity at all concentrations of the nanoparticles compared with the standard ciprofloxacin (Bacteria) and fluconazole (Fungi) were revealed by ANOVA and SPSS statistical tools during sensitivity testing of organisms (Table 3). Despite the fact that none of the as-synthesized nanoparticles was able to compete with ciprofloxacin and fluconazole (standards) in terms of activity, Minimum Inhibitory Concentration (MIC), Minimum Bactericidal Concentration (MBC) and Minimum Fungicidal Concentration (MFC) tests showed the activities of Ag and Ag/Ni nanoparticles on S*. aureus, S. pyogenes, E. coli, P. aeruginosa, C. albicans* and *T. rubrum* (Table 4). This is depicted in one-way analysis of variance (ANOVA) using SPSS statistical tool (significance at P < 0.05).

## 2 Experimental design, materials and methods

### 2.1 Plant collection

**Fig. 3:**
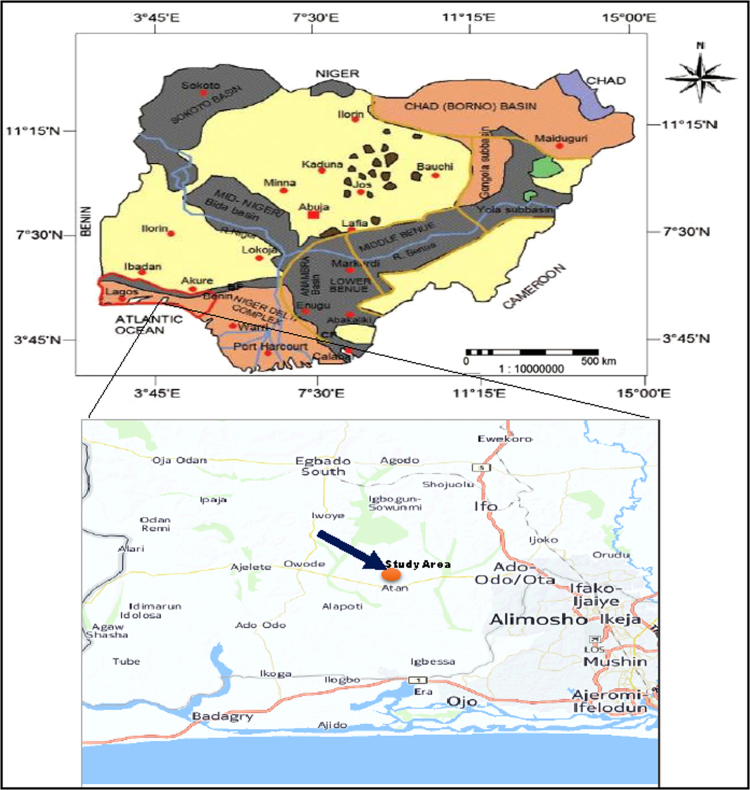
Map of Nigeria with arrow showing plant collection site.

*Canna indica* plant was collected from gardens at Atan-Iju, Ogun State, Nigeria. The collection site is located in moderately hot, humid tropical zone of Southwest Nigeria (Fig. 3).

### 2.2 Ag and Ag/Ni bimetallic nanoparticles syntheses

Plant extract (20 mL) was added to 200 mL of the varied equal molar concentrations (0.5–3.0 mM) of precursor mixture solution (100 mL AgNO_3_ and 100 mL Ni(NO_3_)_2_.6H_2_O) in a beaker. The reaction mixture was continuously stirred with gradual heating to 70 °C on a hot plate [2], [3]. Ag NPs and Ag/Ni bimetallic nanoparticles were collected separately by centrifugation using centrifuge model 0508-1; operated at 5000 rpm for 30 min. For purification, suspension from monometallic nanoparticles was re-dispersed in distilled deionized water so as to remove the unbounded organics, and finally centrifuged at 5000 rpm for 10 min. This same process was repeated for Ag/Ni bimetallic nanoparticles. Monometallic nanoparticles have been synthesized using chemicals like polyols matrix among others [4]; however, in this study, aqueous extract of *Canna indica* served as reducing agent instead of toxic chemicals in the syntheses of Ag NPs of an average diameter of 9.10 ± 1.12 nm and Ag/Ni hybrid nanoparticles of 9.86 ± 2.37 nm mean diameter from TEM analysis [1]. Data generated from energy-dispersive X-ray spectrometer (EDX) also supported formation of Ag NPs and Ag/Ni bimetallic nanoparticles as revealed in Table 1, Table 2. It is worth stating that only plant extracts that are rich in phytochemicals can reduce metal precursor. Hence, phyto-reduction process could be used as an analytical tool to screen plants rich in vitamins and biomolecules.

**Table 1:** The EDX data of Ag nanoparticles.

Element	Series	[wt%]	[norm. wt.%]	[norm. at%]	Error in %
Silver	L-series	55.44	87.58	89.25	0.77
Oxygen	K-series	1.35	3.99	6.13	1.11
Carbon	K-series	1.14	5.25	3.38	0.36
Nickel	K-series	0.96	1.60	0.62	0.09
Cobalt	K-series	0.93	1.56	0.60	0.09
Phosphorus	K-series	0.01	0.02	0.01	0.00
Sulphur	K-series	0.00	0.00	0.00	0.00
	Sum:	59.83	100.00	100.00

**Table 2:** The EDX data of Ag/Ni bimetallic nanoparticles.

Element	Series	[wt%]	[norm. wt.%]	[norm. at%]	Error in %
Silver	L-series	33.33	49.90	69.22	0.80
Oxygen	K-series	10.11	12.11	8.12	5.85
Carbon	K-series	14.21	16.20	9.96	0.38
Nickel	K-series	14.45	19.46	11.11	0.60
Cobalt	K-series	0.55	0.32	0.46	0.06
Phosphorus	K-series	1.12	1.11	0.01	0.09
Sulphur	K-series	0.02	0.90	1.12	0.04
	Sum:	73.79	100.00	100.00	

### 2.3 Optical property

Maximum absorption wavelength was measured by placing each aliquot sample taken at time intervals in quartz cuvette operated at a resolution of 1 nm, distilled-deionized water was used as reference solvent. Data was collected from a double beam thermo scientific Genesys 10S UV–vis spectrophotometer; used to determine the optical property, carried out between 200–800 nm wavelength ranges (Supplementary Tables 1 and 2 are the data for growth comparison in Ag/Ni and Ag nanoparticles respectively synthesized at 70 °C, 30 min).

### 2.4 Determination of antimicrobial activity of the synthesized nanoparticles / Sensitivity of test organisms / Agar well diffusion method

Antimicrobial properties of the biosynthesized nanoparticles were investigated in the form of sensitivity testing, using the modified version of the method described in literature [5], [6]. The procedure was in agreement with recommended standards of National Committee for Clinical Laboratory Standards (NCCLS) [7]. Antimicrobial activity was determined by measuring the zone of inhibition around each well (excluding the diameter of the well) for each nanoparticle obtained from the plant extract (Table 3). Duplicate tests were conducted against each organism.

**Table 3:** Sensitivity testing of organisms with standard deviation in zones of inhibition (Agar Diffusion Test).

Nanoparticles	Organisms/Mean zone diameter (mm)±SD					
	*Staphylococcus aureus*	*Streptococcus pyogenes*	*Escherichia coli*	*Pseudomonas aeruginosa*	*Candida albicans*	*Trichophyton rubrum*
Ag 0.5	7±0.2	9±0.4	7±0.1	Nil	9±0.2	Nil
Ag 1.0	8±0.5	12±0.8	9±0.2	Nil	12±0.3	7±0.2
Ag 2.0	10±0.4	15±0.5	13±0.4	7±0.2	13±0.4	8±0.1
Ag 3.0	13±1	14±0.2	15±0.3	8±0.1	15±0.2	9±0.1
***STAT***	***P>0.05***	***P>0.05***	***P>0.05***	***P>0.05***	***P>0.05***	***P>0.05***
Ag/Ni 0.5	9±0.2	11±0.4	12±0.5	8±0.3	8±0.1	9±0.2
Ag/Ni 1.0	12±0.3	14±0.6	10±0.2	9±0.2	10±0.2	8±0.1
Ag/Ni 2.0	13±0.1	15±0.2	14±0.4	8±0.1	11±0.2	12±0.3
Ag/Ni 3.0	15±0.4	16±0.6	17±0.6	9±0.1	12±0.1	16±0.2
**STAT**	**P<0.05**	**P<0.05**	**P<0.05**	**P<0.05**	**P<0.05**	**P<0.05**
Control	21±0.8	18±0.3	21±0.2	20±0.4	19±0.6	18±0.3
***STAT***	***Control vs Aa, Ba, Ca, Da, Ea, A, B, C, E, F-value 21.45, P<0.05***					

Control -Ciprofloxacin (Bacteria) and Fluconazole (Fungi), mean zone inhibition (mm) ± standard deviation of three replicates [1].

Ag 0.5=Silver nanoparticles formed by reducing 0.5 mM AgNO_3_ concentration solution, Ag 1.0 = Silver nanoparticles formed by reducing 1.0 mM AgNO_3_ concentration solution, Ag 2.0=Silver nanoparticles formed by reducing 2.0 mM AgNO_3_ concentration solution, Ag 3.0=Silver nanoparticles formed by reducing 3.0 mM AgNO_3_ concentration solution, Ag/Ni 0.5=Silver/nickel bimetallic nanoparticles formed by reducing 0.5 mM precursor mixture solution Ag/Ni 1.0= Silver/nickel bimetallic nanoparticles formed by reducing 1.0 mM precursor mixture solution, Ag/Ni 2.0 = Silver/nickel bimetallic nanoparticles formed by reducing 2.0 mM precursor mixture solution, Ag/Ni 3.0 = Silver/nickel bimetallic nanoparticles formed by reducing 3.0 mM precursor mixture solution. *C. indica* leaf extract was the reducing agent.

### 2.5 Determination of Minimum Inhibitory Concentration (MIC) by Tube Dilution Method

Sterile test tubes (12) were arranged in a rack. 1 mL of sterile nutrient broth was added to tube labelled 2 to 10. Known nutrients broth concentration (1 mL) was added to tubes 1 and 2. Afterwards, serial doubling dilution from tube 2 to tube 10 was made, while the remaining 1 mL was discarded. 1 mL of ciprofloxacin was added to tube 11 (positive control); and water to tube 12 (negative control). 1 mL of 0.5 McFarland was added overnight and broth culture to all the tubes and then covered. The experiment was incubated overnight at 37 °C and observed for the highest dilution showing no turbidity. The zone of inhibition was then verified and interpreted according to CLSI guidelines [8] and the MIC was determined (Table 4).

**Table 4:** Minimum inhibitory concentration (MIC), Minimum bactericidal concentration (MBC) and minimum fungicidal concentration (MFC).

Nanoparticles		Organisms/MIC, MBC & MFC (mg/mL)					
		*Staphylococcus aureus*	*Streptococcus pyogenes*	*Escherichia coli*	*Pseudomonas aeruginosa*	*Candida albicans*	*Trichophyton rubrum*
		MIC, MBC	MIC, MBC	MIC, MBC	MIC, MBC	MIC, MFC	MIC, MFC
Ag 0.5		100, 100	50, 100	100, 100	100, 100	50, 50	100, 100
Ag 1.0		100, 100	25, 50	50, 100	100, 100	25, 25	100, 100
Ag 2.0		50, 100	12.5, 25	12.5, 25	100, 100	12.5, 25	100, 100
Ag 3.0		12.5, 25	12.5, 25	12.5, 12.5	100, 100	12.5, 12.5	50, 100
***STATISTICS***		***P<0.05***	***P<0.05***	***P<0.05***	***P<0.05***	***P<0.05***	***P<0.05***
Ag-Ni 0.5		50, 100	25, 50	12.5, 25	100, 100	50, 100	50, 100
Ag-Ni 1.0		12.5, 25	12.5, 12.5	25, 50	50, 100	25, 50	100, 100
Ag-Ni 2.0		12.5, 25	12.5, 12.5	12.5, 12.5	100, 100	12.5, 25	12.5, 12.5
Ag-Ni 3.0		12.5, 12.5	6.25 ,12.5	6.25, 12.5	100, 100	12.5, 25	12.5, 25
***STATISTICS***		***P<0.05***	***P<0.05***	***P<0.05***	***P<0.05***	***P<0.05***	***P<0.05***
Control		3.13	6.25	6.25	6.25	6.25	6.25
***STATISTICS***		***Control vs Aa, Ba, Ca, Da, Ea, A, B, C, E, F-value 34.06, P<0.05***					

Control- Ciprofloxacin (Bacteria) and Fluconazole (Fungi) [1].

Ag 0.5=Silver nanoparticles formed by reducing 0.5 mM AgNO_3_ concentration solution, Ag 1.0=Silver nanoparticles formed by reducing 1.0 mM AgNO_3_ concentration solution, Ag 2.0=Silver nanoparticles formed by reducing 2.0 mM AgNO_3_ concentration solution, Ag 3.0 = Silver nanoparticles formed by reducing 3.0 mM AgNO_3_ concentration solution, Ag/Ni 0.5=Silver/nickel bimetallic nanoparticles formed by reducing 0.5 mM precursor mixture solution Ag/Ni 1.0=Silver/nickel bimetallic nanoparticles formed by reducing 1.0 mM precursor mixture solution, Ag/Ni 2.0=Silver/nickel bimetallic nanoparticles formed by reducing 2.0 mM precursor mixture solution, Ag/Ni 3.0=Silver/nickel bimetallic nanoparticles formed by reducing 3.0 mM precursor mixture solution. *C. indica* leaf extract was the reducing agent.

### 2.6 Determination of Minimum Bactericidal Concentration (MBC)

The MBC is the lowest concentration of antibiotic agent that kills at least 99.9% of the organisms. To determine MBC using Doughari modified method [9], 0.5 mL of the sample was removed from those tubes from MIC which did not show any visible sign of growth and inoculated on sterile Mueller Hinton agar by streaking. The plates were then incubated at 37 °C for 24 h. The concentration at which no visible growth was seen was recorded as the minimum bactericidal concentration (Table 4).

### 2.7 Determination of Minimum Fungicidal Concentration (MFC)

The method described by Doughari was used to determine MFC. 0.5 mL of the sample which showed no visible sign of growth during MIC screening was taken from the test tubes, and then inoculated on sterile potato dextrose agar by streaking. The plates were then incubated at 37 °C for 24 h. The concentration at which no visible growth was seen was recorded as the minimum fungicidal concentration (Table 4) [9].
