# Supplementary material for: Dataset on the evaluation of antimicrobial activity and optical properties of green synthesized silver and its allied bimetallic nanoparticles
Source: Data Brief. 2018 Oct 24;21:989–95. doi: 10.1016/j.dib.2018.10.054 (PMC6222069; doi:10.1016/j.dib.2018.10.054)
Supplement: Supplementary file 1 — Supplementary material. [file mmc1.docx]

***COVER LETTER/CONFLICT OF INTEREST ATTESTATION***

*May 29, 2018*

*The Editor-in-Chief*

*Data-in-Brief*

***NO CONFLICT OF INTEREST***

*Dear Sir,*

*This is to notify you that there is no conflict of interest of any kind regarding*

“Dataset on the evaluation of antimicrobial activity and optical properties of green synthesized silver and its allied bimetallic nanoparticles”

*Yours faithfully,*

*Akinsiku, A. A. (Ph.D)*

*Department of Chemistry,*

*Covenant University,*

*Canaan Land,
Nigeria.*
